# Molecular Cloning, Characterization, and Expression of *MiSOC1*: A Homolog of the Flowering Gene SUPPRESSOR OF OVEREXPRESSION OF CONSTANS1 from Mango (*Mangifera indica* L)

**DOI:** 10.3389/fpls.2016.01758

**Published:** 2016-11-29

**Authors:** Junya Wei, Debing Liu, Guoyin Liu, Jie Tang, Yeyuan Chen

**Affiliations:** ^1^Applied Science and Technology College, Hainan UniversityHainan, China; ^2^Tropical Crops Genetic Resources Institute, Chinese Academy of Tropical Agricultural Sciences/National Tropical Fruit Improvement Center/Hainan Tropical Fruit Cultivation Engineering Technology Research CenterHainan, China

**Keywords:** mango (*Mangifera indica* L), MADS-box, MiSOC1, flowering, analysis

## Abstract

MADS-box transcription factor plays a crucial role in plant development, especially controlling the formation and development of floral organs. Mango (*Mangifera indica* L) is an economically important fruit crop, but its molecular control of flowering is largely unknown. To better understand the molecular basis of flowering regulation in mango, we isolated and characterized the MiSOC1, a putative mango orthologs for the *Arabidopsis* SUPPRESSOR OF OVEREXPRESSION OF CONSTANS1/AGAMOUS-LIKE 20 (SOC1/AGL20) with homology-based cloning and RACE. The full-length cDNA (GenBank accession No.: KP404094) is 945 bp in length including a 74 bp long 5′ UTR and a 189 bp long 3′ UTR and the open reading frame was 733 bps, encoding 223 amino acids with molecular weight 25.6 kD. Both sequence alignment and phylogenetic analysis all indicated that deduced protein contained a conservative MADS-box and semi-conservative K domain and belonged to the SOC1/TM3 subfamily of the MADS-box family. Quantitative real-time PCR was performed to investigate the expression profiles of MiSOC1 gene in different tissues/organs including root, stem, leaves, flower bud, and flower. The result indicated MiSOC1 was widely expressed at different levels in both vegetative and reproductive tissues/organs with the highest expression level in the stems’ leaves and inflorescences, low expression in roots and flowers. The expression of MiSOC1 in different flower developmental stages was different while same tissue –specific pattern among different varieties. In addition, MiSOC1 gene expression was affect by ethephon while high concentration ethephon inhibit the expression of MiSOC1. Overexpression of *MiSOC1* resulted in early flowering in *Arabidopsis*. In conclusion, these results suggest that MiSOC1 may act as induce flower function in mango.

## Introduction

Flowering is an important agronomic trait in crops and is the most dramatic transition known as the floral transition changed from vegetative phase to reproductive phase in a life cycle of flowering plant. For achieve reproductive success, determining the optimal flower timing is critical for flowering plants. The transition is controlled precisely using the model plant *Arabidopsis thaliana* by complex flowering regulatory networks in response to various environmental and endogenous signals (Blazquez et al., 2003; Ausin et al., 2005; Wang et al., 2009). The regulatory networks integrate different signals to determine whether promote flower transition or repress flower transition which use this way to determine the progression of flowering. Extensive genetic and physiological analyses have revealed that at least four major genetic pathways (long-day, autonomous, vernalization, and gibberellin-dependent pathways) regulated floral induction of *A. thaliana* (Liu et al., 2012). Several flowering pathway integrators such as FT, SOC1/AGL20, LLFY, and FLC, these gene integrate multiple flowering pathways signals and these genes expression levels eventually determine the exact flowering time (Sung et al., 2003).

MADS-box genes are key components of the networks that control the transition to flowering and flower development. Extensive studies both in dicots and monocots have led to substantial progress in understanding the molecular mechanisms of the process of vegetative to floral transition and flower development. MADS-box genes encode a large family of transcription factors that share a highly conserved DNA-binding domain, the MADS-box domain, which binds to a CC(A/T) 6GG (CArG) box on target genes in plants (Riechmann et al., 1996). MADS-box genes control the identity of the apex meristem, development of lateral organs, and flowering time of plants (Kater et al., 2006). MADS-box genes have been recruited as master regulatory genes that control developmental processes, such as inflorescence and flower formation during plant evolution. MADS-box proteins as key regulators effect on many steps of development and differentiation in the flowering plant (Ng and Yanofsky, 2001). The MADS-box gene family in *Arabidopsis* is necessary for the flowering time regulation, floral meristem identity determinacy, floral tissue/organ growth, fruit development regulation as well as vegetative development regulation (Alvarez-Buylla et al., 2000a,b; Riechmann and Ratcliffe, 2000).

SUPPRESSOR OF OVEREXPRESSION OF CONSTANS1 encodes a MADS-box type II (MIKCC) transcription factor protein in plants and plays a crucial regulatory role during plant development and floral organogenesis (Hepworth et al., 2002; Messenguy and Dubois, 2003). In *Arabidopsis* SOC1 is a critical integrator which encodes a MADS-box protein and promotes flowering which integrates multiple flowering signals and regulates flowering time and floral patterning (Melzer et al., 2008; Liu et al., 2009). SOC1 genes are widely expressed in various tissues/organs including roots, leaves, shoot meristems, and floral organ primordia and its expression levels are tightly controlled by multiple flowering pathways (Borner et al., 2000; Watson and Brill, 2004; Shen et al., 2011).

SUPPRESSOR OF OVEREXPRESSION OF CONSTANS1 (SOC1) is a member of a large family of transcription factors which composed of the MADS-box (M), an intervening (I) region, a keratin (K) box, and a C-terminal domain and is conserved among angiosperm including both monocotyledons plant and dicotyledons plant (Ferrario et al., 2004; Lee et al., 2004; Zhong et al., 2012) and had been isolated from *Arabidopsis*, *Oryza sativa, Petunia hybrida, Citrus sinensisx, Trillium camtschatcense, Triticum aestivum L, Zea mays, Indian Brassica* and *Glycine max [L.] Merr*. (Moon et al., 2003; Tadege et al., 2003; Ferrario et al., 2004; Lee et al., 2004, 2008; Nakamura et al., 2005; Shitsukawa et al., 2007; Ryu et al., 2009; Lee and Lee, 2010; Zhong et al., 2012; Suzhou et al., 2014; Sri et al., 2015). Act as central floral integrators, SOC1 is regulated by the upstream genes CONSTANS (CO) and FLOWERING LOCUS C (FLC). The CO gene acts as a floral activator which mediates the photoperiod pathway of flower plant, whereas the FLC gene acts as a floral repressor which mediates the autonomous and vernalization pathways of flower plant (?). CO primarily activate SOC1 through FT gene while FLC directly binding to its promoter to represses SOC1 expression (Hepworth et al., 2002; Wigge et al., 2005; Yoo et al., 2005; Searle et al., 2006). SOC1 regulates not only flowering time but also mediates other biological processes in plant such as *C. sinensis* floral organ senescence (Tan and Swain, 2007), *Gerbera hybrida* floral organ identity and petal development (Ruokolainen et al., 2011), *G. max* pathogen response (de Sá et al., 2012), *Fragaria vesca* GA biosynthesis (Mouhu et al., 2013), *Hordeum vulgare* seed development (Papaefthimiou et al., 2012), and *Dendrobium* Chao Parya Smile floral meristem development (Ding et al., 2013). Therefore, further research is required to uncover the functional divergence of SOC1s among plant species. However, little is known about the structure, expression of the homologous SOC1 in mango.

Mango (*Mangifera indica* L.) is one of the most important fruit crops in the world and widely grows from the tropics to the subtropics regions including southeastern Asia and Central and South America. It is important to understand floral induction in mango trees to ensure regular flower bud formation for stable production every year. Flowering in mango is a complex process varies greatly from year to year depending on climatic conditions. There have several floral promotion pathways in mango but little information are known on the internal regulation of floral induction in mango. Isolation of mango flowering genes will greatly help in understanding the regulation of floral induction and promotion in mango.

In the present research, a homolog of the flowering gene SOC1, was isolated from the inflorescence tissue by Rapid Amplification of cDNA ends (RACE) of a popular variety of mango (*M. indica* var. Carabao). Then its molecular characterization and phylogenetic evolutionary relationships were investigated. In particular, the transcriptional expression patterns of MiSOC1 in differently tissues/organs and developmental phases in mango were studied. Finally, the functions of MiSOC1 in promoting plant growth and advancing flowering time were demonstrated in *A. thaliana*. Our results suggest that MiSOC1 is a flowering promoter in mango.

## Materials and Methods

### Plant Material and Growth Condition

Ten-year-old (*M. indica*. cv. Carabao) mango trees grafted on ‘Neelum’ rootstocks grown in mango germplasm nursery maintained in Germplasm Research Institute of Tropical Crops of Chinese Academy of Tropical Agriculture Sciences, Hainan, China, were used to isolate flowering-related genes and some later express pattern analysis. Inflorescence tissues at anthesis period were collected and used for flowering-related gene clone. To study the expression of MiSOC1 gene in different tissue of different cultivars, different tissues (root, stem, leaf, flower bud, and flower) at anthesis stages of three mango cultivars (Carabao, Kiett, and jinhuang) were collected and used for tissue specific expression analysis.

To study whether ethephon treatments affected MiSOC1 expression, we designed and applied four different treatments to the inflorescence tissues and the treatments were designed as follows:

(1) Spay 500 × 40% ethephon with 0.1% Tween 20 to the inflorescence tissues.
(1) Spay 1000 × 40% ethephon with 0.1% (v/v) Tween 20 to the inflorescence tissues.
(1) Spay 1500 × 40% ethephon with 0.1% (v/v) Tween 20 to the inflorescence tissues.
(1) Spay with 0.1% (v/v) Tween 20 to the inflorescence tissues as control.

These treatments were repeated three times for 1 week. 0.1% Tween-20 was added to the solutions as a surfactant. Three treatments were used with 10 plants evaluated per treatment. Then 7 days after the above treatments, inflorescence tissues samples were collected for qRT-PCR analysis.

All of the collected samples were frozen with liquid nitrogen immediately and stored at -80°C in a refrigerator until RNA preparation. Each experiment was conducted three biological replicates using a completely random design.

*Arabidopsis thaliana* ecotype Columbia was used to generate transgenic plant. Seeds were sterilized and sowed on half-strength MS (Murashige and Skoog, 1962) medium supplemented with 3% (W/V) sucrose and 6.8% (W/V) agar. The wild-type or transgenic plants were grown in a greenhouse with a 14/10 h light/dark cycle at 22 ± 1°C and 50–70% relative humidity with cool-white fluorescent lights. Seeding were cultured under normal long-day conditions.

### RNA Extraction and First Strand cDNA Synthesis

According to the RNA extraction protocols, total RNA of different samples was extracted using a Qiagen RNeasy plant kit according to the manufacturer’s instructions, followed by DNase I treatment, phenol/chloroform extraction and precipitation with ethanol plus sodium acetate. The concentration and purity quotient of RNA were determined by the measurement of 260 nm absorbance and 260/280 nm absorbance, respectively. The integrity of total RNA was determined by 2% agarose gel electrophoresis and ethidium bromide staining. The resulting RNA preparation was incubated at 70°C for 10 min and snap-cooled on wet ice for 5 min and then used as template for reverse transcription- polymerase chain reaction (RT-PCR). cDNA synthesis was carried out according to manufacturer’s instructions. The synthesized cDNA was kept at -80°C until further use.

### Isolation of Mango MiSOC1 Gene

For cloning the mango MiSOC1 gene, we first isolated partial mango MiSOC1 cDNA fragment with degenerate PCR (polymerase chain action) method, then obtain the full length mango MiSOC1 cDNA sequence with 3′and 5′ RACE. The primer sets are listed in **Table 1** designed based on the 5′ and 3′-terminal sequence of partial mango MiSOC1 sequence obtained in our laboratory. The reaction was performed in an S1000TM thermal cycler (Bio-Rad, USA). The sequencing results of the 3′ and 5′ RACE PCR products were searched against the NCBI database, then determined the splice and clone the full length of MiSOC1gene. The 3′ and 5′ RACE PCR were performed with the protocol of the 3′ and 5′ full RACE Core Set (TaKaRa, Japan). The PCR production was purified using a PCR purification kit (Qiagen), inserted into the pGEM-T Easy Vector (Promega) and transformed into competent *Escherichia coli* DH5a cell. The recombinant plasmids were identified and the positive clones were picked and sequenced in Shanghai Sangon Biological Engineering Technology and Services Co., Ltd (Shanghai China). The confirmed full-length cDNA of MiSOC1was was deposited at GenBank (accession number KP404094) and used for molecular characterization and bioinformatics analysis later.

**Table 1:** Sequences of primers used in this study.

Primer name	Sequence (5′ → 3′)	Application
3-O	GCAAAGAGAAGAAATGGGCTG	3′ RACE
3-I	TCTGTTCTTTGTGATGCTGAGGT	
5-O	TTCCACCACACTTCTCAGCCAGCCTTA	5′ RACE
5-I	ATGGTAAGGGCAACCTCAGCATCACAA	
MiSOC-1	GGAATTCGACAGAAAGAGTTGGGGTG	Amplifying the full-length coding region
MiSOC-2	GCTCTAGACTATTTGGCTAGTAGTCAAAAT	
ActinF	CGTAGCACCAGAAGAACA	qRT-PCR
ActinR	CATAAAGGGAGAGGACAG	
MiSOC-3	ACAGCGTAAGCAACATTCG	qRT-PCR
MiSOC-4	CCACCACACTTCTCAGCCA	


### Sequence Comparison and Phylogenetic Analyses

Both the nucleotide sequence and the amino acid sequence of mango MiSOC1 were analyzed on the NCBI blast program^1^. The conserved domain of mango MiSOC1 sequence was predicted by the motif scan analysis program^2^. The multiple sequence alignments of the mango MiSOC1 amino acid with other plant SOC1 protein sequence were conducted by using the Clustal W program and Genedoc program. Molecular weight and the theoretical isoelectric point (pI) were calculated using the PeptideMass program^3^. The potential sequence motifs were identified using the ISREC web server^4^. Protein sequences were aligned using ClustalW (Thompson et al., 1994), and the alignment was edited with BioEdit^5^. The deduced amino acid sequence of MiSOC1 was used as a query to search the protein database from the NCBI. Through sequence comparison and identified, 22 protein sequences which have more than 68% identity with MiSOC1 were selected to construct a phylogenetic tree by MEGA 5.0 software (Tamura et al., 2011) using Neighbor-Joining (NJ) method (Saitou and Nei, 1987), and the bootstrap test was carried out with 1,000 bootstrap replicates. The numbers at each node represent the bootstrap support (percentage).

### RT-PCR and Real-Time Quantitative PCR

Total cDNA was used as the template for RT- and q-PCR. For semi-quantitative PCR, first-strand cDNA was prepared from different tissues (root, stem, leaf, flower bud, and flower) of mango plants according to the manufacturer’s protocol of PrimeScript II 1st Strand cDNA Synthesis Kit and stored at -20°C. The PCR reactions were carried out using Amplitaq DNA polymerase (Roche, Mannheim, Germany) according to the manufacturer’s instructions. The PCR program consisted of 95°C for 5 min, followed by 36 cycles of 94°C for 45 s, 55°C for 45 s, and 72°C for 1 min, and a final extension at 72°C for 10 min. β-actin expression of different tissues was used as the internal control to normalize the results of semi-quantitative PCR analysis.

The tissue-specific expression of mango MiSOC1 transcript was determined by qRT-PCR using the gene-specific primers (**Table 1**). β-actin expression of different tissues was also used as the internal control for the normalize of the results of qRT-PCR analysis. The efficiency and specificity of the primers were detected before performing the qRT-PCR analyses process. Each reaction was performed in triplicates. The reactions were carried out in a volume of 10 μL, including 0.4 μL of each primer (10 μM), 3.5 μL of PCR-grade water, 0.8 μL cDNA, 0.1 μL of ROX Reference Dye (50×), and 10 μL of SYBR premix Ex Taq II (Perfect Real Time; TaKaRa, Dalian, China) on a Stratagene Mx3005P quantitative PCR machine. Each reaction was performed in triplicates. The program contained one cycle of 95°C for 5 min, 40 cycles of 95°C for 5 s, 60°C for 30 s, followed by one cycle of 95°C for 30 s, 60°C for 30 s. At the end of the qRT-PCR amplified reactions, the melting curve analysis was implemented to confirm the credibility of each qRT-PCR analysis. To identify the PCR products a melting curve was performed from 65 to 95°C with observations every 0.2°C and a 10-s hold between observations. Every sample was analyzed in triplicate to certify the repetitiveness and credibility of experimental results. The qRT-PCR results were measured by using 7500 SDS software (Applied Biosystems, USA) with 2^-ΔΔ^*^C^*^t^ methods (Schmittgen and Livak, 2008). The relative expression levels of mango MiSOC1 transcript were determined in the tissue-specific expression analysis. The expression levels of mango β-actin gene as the reference gene with ActinF/ActinR as primers were quantified in parallel with the target genes as an internal control. Three biological samples and triplicate qRT-PCR reations for each combination of primers and sample were analyzed.

### Constructing Expression Vectors and Plant Transformation

The ORF of the MiSOC1 gene was cloned into the binary plant transformation vector pCAMBIA1301 (CAMBIA, Canberra, Australia) under the control of the Cauliflower mosaic virus (CaMV) 35S promoter using the primer pair: 5′- CCATGGAATTCGACAGAAAGAGTTGGGGTG -3′ (NcoI site underlined) and 5′- AGATCTAGACTATTTGGCTAGTAGTCAAAAT -3′ (BglII site underlined) and re-amplified and re-sequenced to confirm the clone sequence. Then the recombinant plasmid was transformed into *A. thaliana* ecotype Columbia plants using the floral dip method (Clough and Bent, 1998) mediated by *Agrobacterium tumefaciens* strain EHA105. The homozygote plants were selected for further analysis.

The T3 transgenic and wild-type *Arabidopsis* were directly planted in nutritive soil. qRT-PCR was used for gene expression assays analyze of *MiSOC1* in transgenic *Arabidopsis* when the seedlings were harvested at 10 days after the germination. The total number of rosette leaves was counted for analysis of the flowering time when these *Arabidopsis* began floral bolting, and 30 transgenic plants and wild-type *Arabidopsis* were counted, respectively.

## Results

### Isolation, Identification, and Sequence Analysis of MiSOC1

An EST which showed high sequence similarity compared with SOC1 gene through BLAST search method in NCBI database search was gained with degenerate primer RT-PCR in our laboratory. Based on the sequence of EST, specific 3′ and 5′ RACE primers were designed for cloning the full length candidate gene. By RACE procedure, the unknown sequences for the 5′ and 3′ ends of mango MiSOC1 were amplified successfully from the mango inflorescence tissues, respectively. The amplification with primer pair MiSOC-1/ MiSOC-1 spanning the coding region yielded 733 bp bands, termed mango MiSOC1.

SUPPRESSOR OF OVEREXPRESSION OF CONSTANS1 act as a main integrator, the structure and function of SOC1 is highly conserved in almost all plant species. Using the 3′ and 5′ RACE approach, a MADS box gene, *MiSOC1* was cloned. The result of sequence alignment analysis indicated that *MiSOC1* was a homolog gene of MADS-box SOC/TM3 family. Both the complete nucleotide and deduced amino acid sequences of mango MiSOC1 were all shown in **Supplementary Figure 1**. Deduced amino acid sequence of the mango MiSOC1 showed high identity (99%) to that from *M. indica*. cv. Siji, moderate identity (72–74%) to other plant SOC1 such as *Litchi chinensis* and *C. sinensis* (data not shown). The full-length cDNA of mango *MiSOC1* sequence was 945 bp with a 74 bp 5′ UTR, a 189 bp 3′ UTR and a of 672 bp ORF. There also have a putative polyadenylation signal sequence (AATAAA) at 790 bp of mango *MiSOC1* predicted nucleotide sequence, which was 130 bp upstream from the poly (A) tail (**Supplementary Figure 1**).

The coding DNA sequence (CDS) of mango MiSOC1 encoded a polypeptide of 223 amino acids with an estimated molecular mass of 25.69 kDa and a predicted isoelectric point (pI) of 8.96. Based on the conserved domain of mango MiSOC1 sequence, the characteristics and domain structure of the predicted amino acid sequence revealed that mango MiSOC1 was a member of the MADX-box superfamily. Both the nucleotide sequence and the amino acid sequence of mango MiSOC1 were analyzed on the NCBI blast program^6^. GenBank analysis revealed that the mango MiSOC1 amino acid sequence showed the closest homology to that of SiJi *M. indica* L (Accession No. ADX97324.1) with 99% identity, followed by the next two close amino acid sequences from *C. sinensis* (Accession No. XM_015534162.1) with 81% identity and *L. chinensis* (Accession No. KC877998.1) with 84% identity.

**FIGURE 1:**
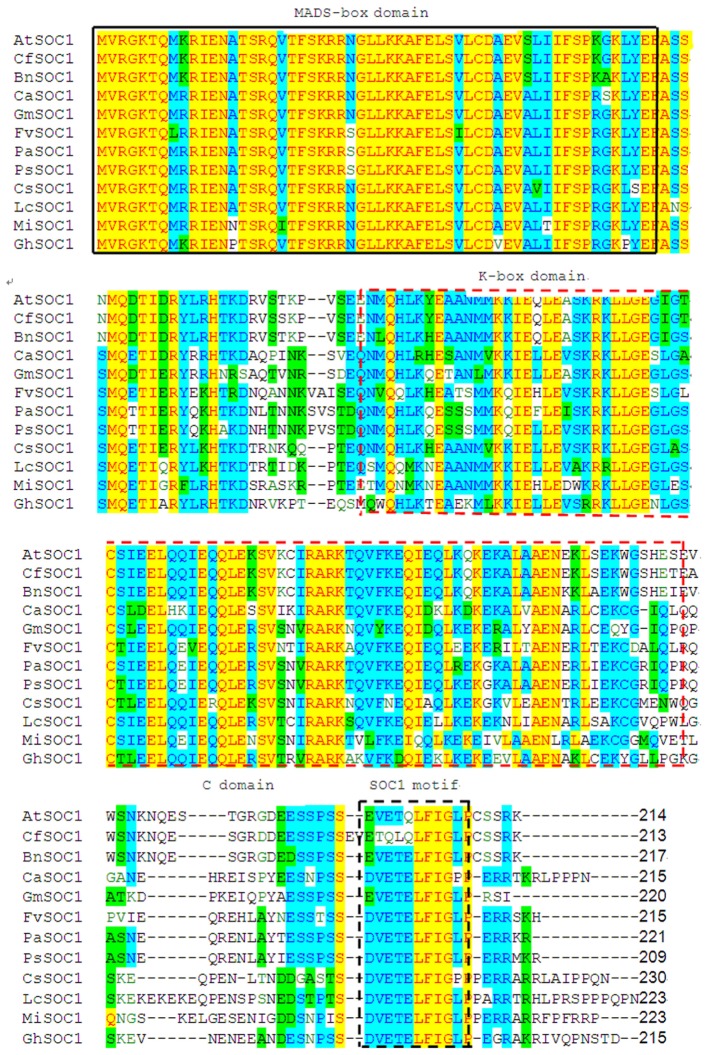
**Alignment of *MiSOC1* predicted amino acid sequence with other *SOC1* protein showing MIKC domains.** Conserved domains (M, K) were boxed with black solid and red dashed line, respectively, while C-domain was located behind K-box. In C-domain, the SOC1 motif is highlighted with black dashed line box. The protein sequences of *SOC1* genes aligned in this study were retrieved from NCBI. The GenBank accession numbers of 11 *SOC1* genes were *LcSOC1*(AGS32267.1), *CsSOC1*(NP_001275772.1), *BnSOC1*(NP_001303107.1), *CaSOC1*(AHI85950.1), *GhSOC1*(AEA29618.1), *PsSOC1*(AGD88523.1), *PaSOC1*(ACO40488.1), *GmSOC1*(NP_001236377.1), *CfSOC1*(AGN29205.1), *AtSOC1*(NP_182090.1) and *FvSOC1*(AEO20231.1).

So far MADS-box genes were isolated from dicots, monocots, and gymnosperms of plant species. The predicted amino acid sequence of MiSOC1 was compared with 11 SOC1 proteins from different species including *L. chinensis*, *C. sinensis*, *Carya cathayensis*, *A. thaliana*, and *F. vesca*. The conserved domain predicted and the motif scan analysis of mango MiSOC1 sequence revealed that there have two potential functional domains in mango MiSOC1 amino acid sequence: MADS_MEF2_like domain (3–77 amino acids) at N terminal and K-box domain (75–170 amino acids) at middle of the protein. The result of multiple alignment analysis make clearly that the domain structure of mango MiSOC1 protein was similar with the other plant SOC1 proteins which belongs to the MADS-box transcription factor (MIKC type) (**Figure 1**). The sequence of MiSOC1 has 52% identity with the sequence of *Arabidopsis* SOC1 and has higher identity with the sequence of other monocots SOC1 gene. The result of multiple sequence alignment revealed a well-conserved SOC1 motif with 11 amino acid residues at their C-termini (**Figure 1**). This motif is a specific characteristic in the TM3 clade of MADS-box genes both in gymnosperms and angiosperms.

It was worth nothing but the three amino acid residues (24th R, 34th E, and 113th G) in MiSOC1 sequence were the same with AGL20/SOC1 from *Arabidopsis*. AGL20/SOC1in *Arabidopsis* is a floral activator and the substitutions of three amino acid residue could affect early flowering time (Lee et al., 2000, 2008). The over-expression of SOC1gene with the 24th R replaced with K suppressed the early flowering in *Arabidopsis*, while over-expression of SOC1 gene with the 34th E replaced with K (or 113th G replaced with E) partially suppressed early flowering in *Arabidopsis*. Therefore, it is possible that MiSOC1 have the similar functions with AGL20/SOC1 gene of *Arabidopsis*. More interestingly, three amino acid residues (2th V, 6th L, and 9th G) are identical in all SOC1 proteins examined which belong to the aliphatic group of amino acids in the SOC1 motif (**Figure 1**). Ding et al. (2013) considered that similar structure and general chemical characteristics of SOC1 proteins may contribute to the specific function in plants and should to be investigated further. The sequence comparison revealed that MiSOC1 belonging to plant type II MADS box genes showed extensive similarity with MADS box proteins. Further analysis of the deduced mango MiSOC1 amino acid sequence by using the SignalP 4.0 server^7^ predicted no signal peptide. Predict the sub-cellular localization for MiSOC1 using ProtComp indicate that the protein is possibly located both cytoplasm and nucleus. In addition, a number of predicted motifs for nuclear localization signals and phosphorylation and glycosylation sites were present in the deduced amino acid sequence.

### MiSOC1 Encodes a MADS-Box Transcription Factor

**FIGURE 2:**
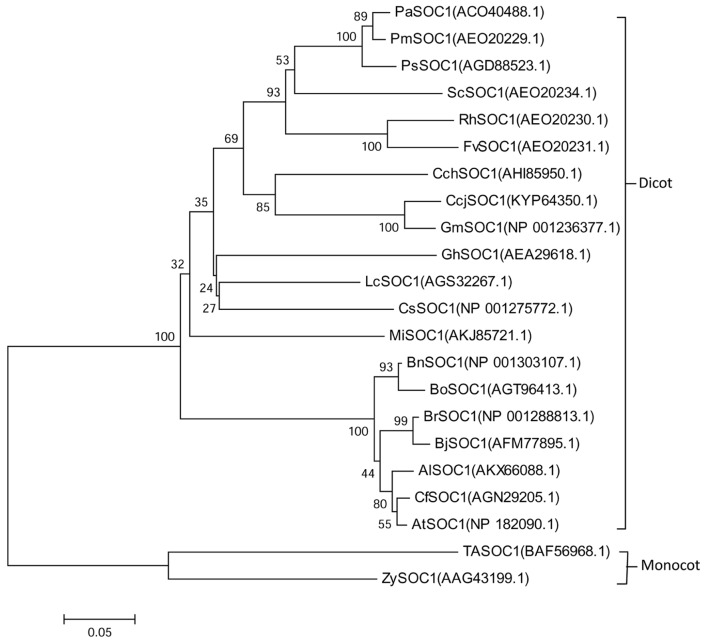
**Phylogenetic analysis of *MiSOC1* and its homologous sequences from various plant species.** The proteins were initially aligned using Clustal W and were used for phylogenetic analysis using MEGA version 6.0 software. The phylogenetic tree was constructed using the neighbor-joining method. Bootstrap percentages were shown at dendrogram branch points. The accession numbers of the genes are *PaSOC1*(ACO40488.1), *PmSOC1*(AEO20229.1), *PsSOC1*(AGD88523.1), *ScSOC1*(AEO20234.1), *RhSOC1*(AEO20230.1), *FvSOC1*(AEO20231.1), *CchSOC1*(AHI85950.1), *CcjSOC1*(KYP64350.1), *GmSOC1*(NP001236377.1), *GhSOC1*(AEA29618.1), *LcSOC1*(AGS32267.1), *CsSOC1*(NP001275772.1), *MiSOC1*(AKJ85721.1), *BnSOC1*(NP001303107.1), *BoSOC1*(AGT96413.1), *BrSOC1*(NP001288813.1), *BjSOC1*(AFM77895.1), *AlSOC1*(AKX66088.1), *CfSOC1*(AGN29205.1), *AtSOC1*(NP182090.1), *TaSOC1*(BAF56968.1) and *ZySOC1*(AAG43199.1).

The conserved domain predicted and the motif scan analysis revealed that there have a strongly conserved MADS_MEF2_like domain and K-box domain at middle of MiSOC1 protein sequence (**Figure 1**). Therefore, it belongs to the MIKC type MADS-box transcription factor. To investigate the evolutionary relationship between MiSOC1 and other SOC1-like genes, a phylogenetic tree was constructed using their amino acid sequences in their MADS-box domain. The phylogenetic tree indicated that all members could be divided into dicot and monocot clades, with MiSOC1 protein belonging to the dicot clade (**Figure 2**). It was found that the whole phylogenetic tree has family specificity. The SOC1 and SOC1-like proteins (22 in total) were clustered together, corresponding to *Rosaceae, Juglandaceae, Fabaceae, Malvaceae, Sapindaceae, Brassicaceae Triticeae*, and *Andropogoneae*. MiSOC1 was closely related to *Sapindaceae* SOC1. These results confirmed that MiSOC1 was a MADS-box gene and a SOC1 homolog in mango.

**FIGURE 3:**
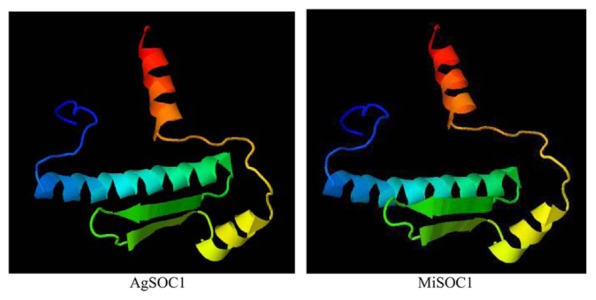
**3D structural model of MADS-box domain of the MiSOCl protein**.

To further characterize MiSOC1, a three-dimensional structure was modeled based on AtSOC1, using Phyre2 batch processing (Kelley et al., 2015). Forty-one percentage of the residues in MiSOC1 were modeled with 100.0% confidence, using the single highest scoring template. The monomer of GmSOC1-like appears to be formed by two spatially distinct domains: a helix-turn-turn-helix structure for the MADS box domain (aa 13–70; **Figure 3**), and a large helical K-box and C-terminal domain, respectively. The MADS box domain of MiSOC1 forms a abba motif that is the same as that of AtSOC1 (**Figure 3**). There have 66.4% α- helix are uniformly distributed in the entire polypeptide chain and 35.4% β-strand or coil folding relatively orderly inlaid in the α- helix, 17.0% β-turn irregular mosaic distribution in the polypeptide chain. Bioinformatic analysis stated clearly that MiSOC1 possessed a highly conserved MEF2-like MADS domain which has the sequence-specific DNA binding ability. Research suggested the K domain which was moderately conserved has been shown to be important for protein–protein interactions. The carboxyl-terminal (C) region which was poorly conserved in sequence may act as a *trans*-activation domain (Riechmann and Meyerowitz, 1997). These analysis results indicated that MiSOC1 protein may function as a mango MADS box transcription factor which functions similar to other SOC1 proteins in plants.

### Expression Patterns Analyses of MiSOC1 in Different Tissues/Organs of Different Varieties, and Different Reproductive Development Stages

**FIGURE 4:**
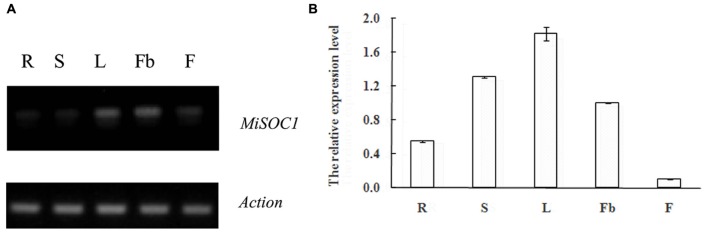
**Semi-quantitative RT-PCR (A)** and Quantitative real-time RT-PCR **(B)** expression of *MiSOC*1 in different tissues of *Mangifera indica*. Carabao is shown. R: root; S: stem; L: leaves; Fb: flower buds; F: flowers. Total RNA was extracted from the indicated organs and RT-PCR was performed. Mango β-action was used as the endogenous control. The levels in roots were arbitrarily set to 1. Error bars represent the standard deviations of three technical PCR replicates.

SUPPRESSOR OF OVEREXPRESSION OF CONSTANS1 gene encodes a MADS box protein which is conserved among plant. In order to get an insight into the potential function of MiSOC1 in mango, we first examined the spatial expression patterns of MiSOC1 in various tissues/organs of mango (*M. indica* var. Carabao) under normal conditions using semi-quantitative RT-PCR and quantitative RT-PCR methods. Total RNA was isolated from various tissues/organs, including roots, stem, leaves, flowers bud, and flower using mango actin gene as an endogenous control. The results showed that the mango MiSOC1 gene displayed distinct tissue-specific patterns in different tissues/organs examined and ubiquitously expressed in mango tissues/organs including root, stem, leaf, flower bud, and flower. The expression of SOC1 in *Arabidopsis* was displayed in leaves, roots and stem meristems (Lee and Lee, 2010). Similarly, MiSOC1 showed the highest expression in leaves, followed by stems, roots and flower bud, less in flowers (**Figure 4**).

Research found that SOC1 act as a general regulator in plant organogenesis development and is a multifunctional protein which regulates flowering time, floral patterning and floral meristem determination in many species (Melzer et al., 2008; Liu et al., 2009). These results indicate that MiSOC1 may act as a floral inducer, closely associated with the transition from vegetative growth to reproductive development of *M. indica* Carabao.

**FIGURE 5:**
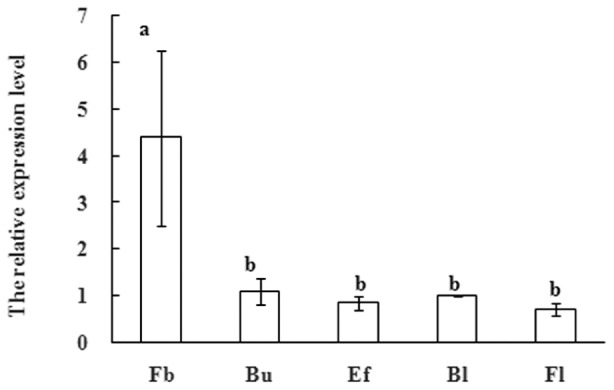
**Expression pattern of *MiSOC1* at flowering stage of *Mangifera indica*.** Carabao is shown. Fb: Flower bud, Bu: bud, Ef: early flower, Bl: blossom; Fl: Fruitlet. The relative expression ratio of each sample is compared to the control group which was blossom, respectively, and arbitrarily set to 1. Different letters represent significant differences at *P* < 0.05 according to Duncan’s multiple range tests. Error bars represent the standard deviations of three technical PCR replicates.

SUPPRESSOR OF OVEREXPRESSION OF CONSTANS1 have also been shown to affect flower development in several plants. After the floral transition in *Arabidopsis*, SOC1 expression remains at an appropriate level in emerging floral meristems, which is regulated by another floral meristem identity gene APETALA1 (Liu et al., 2007). These observations indicate that SOC1-like genes also mediate the subtle development of floral organs. In order to further research function of MiSOC1 during the reproductive development process of mango, quantitative real-time expression analysis was carried out to examine the expression of MiSOC1 in different reproductive development stage of *M. indica* var. Carabao. MiSOC1 expression was detected across all reproductive developmental stages (**Figure 5**). The highest expression levels were observed in floral buds with 4.39-fold higher relative expression compared to the blossom, and the least expression was observed at fruit set which was only 4.39-fold compared to the blossom. These discrepancies suggest that MiSOC1 may participate in the initiation and maintenance of flowering in mango.

**FIGURE 6:**
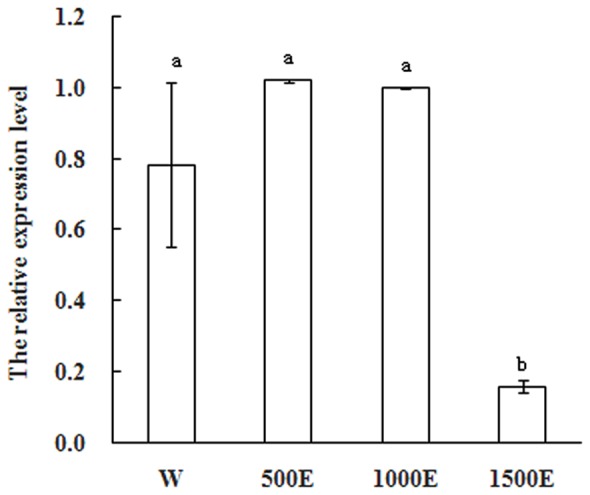
**Expression pattern of *MiSOC1* in different tissues of *Mangifera indica*.** Kiett **(A)** and Jinhuang **(B)** is shown. R: root; S: stem; L: leaves; Fb: flower buds; F: flowers. Total RNA was extracted from the indicated organs and RT-PCR was performed. Mango β-action was used as the endogenous control. The levels in Flower bud were arbitrarily set to 1. Error bars represent the standard deviations of three technical PCR replicates.

Real-Time Quantitative PCR analysis was further performed to detect the expression patterns of mango MiSOC1 gene in various tissues between different varieties under normal conditions. Mango varieties (Kite and jinhuang) were used for tissue specific expression analysis. As for Kiett mango, the highest expression levels were observed in leaves with 13.14-fold higher relative expression compared to the flower bud, and the least expression was observed at blossom which was only 0.12-fold compared to the flower bud (**Figure 6A**). As for Jinhuang mango, the highest expression levels were observed in leaves with 11.57-fold higher relative expression compared to the flower bud, and the least expression was observed at blossom which was only 0.06-fold compared to the flower bud (**Figure 6B**). The results showed that the mango MiSOC1 gene displayed similiar tissue-specific patterns among these three varieties. MiSOC1 expression was detected in reproductive parts of mango at relatively low levels, including in flower bud, flower and fruitlet, whereas its expression in vegetative organs, such as root, stem and leaves significantly increased, with the highest level in leaves. These discrepancies indicate that MiSOC1 may involve in the launch and maintenance of mango flowering process. MiSOC1 function might be closely associated with the transform from vegetative development to reproductive development of mango.

### Expression Analyses of MiSOC1 Response to Different Concentration Ethephon Treatments in Inflorescence Tissues

**FIGURE 7:**
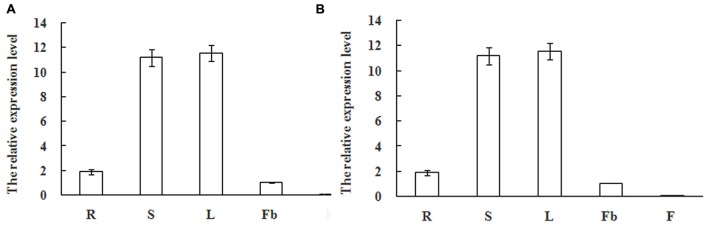
**Expression pattern of *MiSOC1* in different tissues of *Mangifera indica*.** Carabao under ethephon treatment is shown. R: root; S: stem; L: leaves; Fb: flower buds; F: flowers. Total RNA was extracted from the indicated organs and RT-PCR was performed. Mango action was used as the endogenous control. The levels in flower bud with water were arbitrarily set to 1. Different letters represent significant differences at *P* < 0.05 according to Duncan’s multiple range tests. Values are the average of three experiments with three biological replicates with three technical replicates.

Exogenous applications of plant growth regulators such as ethylene have been shown to modify plant architecture that resulting in increased commercial crop growth and production. Ethephon decomposes to ethylene within plant tissues and it is widely used as a plant growth regulator to reduce stem elongation, increase lateral branching, and manipulate flower initiation (Campos et al., 2009). In most mango varieties flowering is not normally profuse. Flowering cannot yet be predicted accurately before floral induction and it is the time to apply ethephon. To further explore whether MiSOC1 expression was regulated by ethephon, mango inflorescence tissues were treated with different concentration ethephon and samples were harvested after 7 days of treatment. As shown in **Figure 7**, the expression level of MiSOC1 transcripts under ethephon treatment increased by 500 × 40% ethephon treatment and reached the higher level by 1000 × 40% ethephon treatment, but repressed by 1500 × 40% ethephon treatment. Taken together, these results revealed that MiSOC1 gene expression was affect by ethephon while high concentration ethephon inhibit the expression of MiSOC1. Clearly, this will be an important clue for future investigation.

### Expression and Function Analysis of *MiSOC1* in Transgenic *A. thaliana* Plants

**FIGURE 8:**
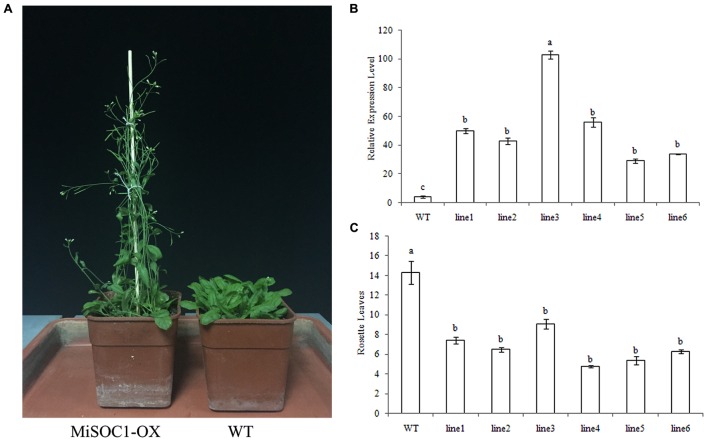
**Overexpression of MiSOC1 promotes flowering in Arabidopsis in Long-day condition and has fewer leaves.** **(A)** The phenotype of MiSOC1 transgenic lines under Long-day condition. **(B)** Transgenic plants were confirmed by qRT-PCR with β-action as an internal reference. Values represent the means, and the error bars represent standard errors for three independent experiments. Different letters represent significant differences at *P* < 0.05 according to Duncan’s multiple range tests. **(C)** The number of rosette leaves during flowering in transgenic plants and wild-type plants.

The SOC1 have been widely isolated from plant and their conserved function is characterized as integrators of multiple flowering signals and flowering promoter. To determine if MiSOC1 had a role in regulating flowering in mango development, transgenic *Arabidopsis* plants that overexpressed the sense constructs of MiSOC1 driven by the CaMV 35S promoter were generated. The transgenic plants were analyzed by qRT-PCR to confirm the presence and expression level of the transferred MiSOC1gene. The result showed that the significantly higher expression levels of MiSOC1 were significantly increased in all the transgenic lines compared to those of wild-type (**Figure 8B**). The independent T3 lines were selected for flowering time analysis under Long-day conditions. The transgenic *Arabidopsis* plants grew very quickly and had a large biomass when they reached flowering. Compared to the wild-type, the transgenic lines significantly promoted flowering under Long-day conditions (**Figure 8A**). Overexpressing *MiSOC1* transgenic plants significantly promoted *Arabidopsis* growth and advanced flowering time which started bolting ranged from 6.3 to 9.1 rosette leaves compared to 14.3 for wild-type plants (**Figure 8C**). In addition, the transgenic lines showed no obviously morphological changes. These results suggested that MiSOC1 acted as a flowering activator in transgenic *Arabidopsis* and could have a positive role in regulation flowering of mango.

## Discussion

Flowering is pivotal for the reproductive behavior of plants, and it is regulated by complex and coordinated genetic networks. SOC1 integrates multiple flowering signals to regulate floral transition in *Arabidopsis*. Precise control of flowering time is an essential developmental process that determines reproductive success of flowering plants. The MADS-box proteins are an important class of transcription factors involved in the regulation of numerous genes with a diverse range of biological functions. Over the last two decades, many homologous SOC1 genes have been cloned and the corresponding SOC1 gene functions were verified (Borner et al., 2000; Lee et al., 2000; Zhong et al., 2012). Although Mango (*M. indica* L.) is one of the most important fruit crops in the world and widely grows from the tropics to the subtropics regions, the molecular mechanisms underlying the floral transition in this family are largely unknown.

In this work, we cloned a new MADS box gene, *MiSOC1* from *M. indica*. Carabao. Similar to other known MADS-box genes, the deduced amino acid sequences from the mango MiSOC1 protein were aligned with other plant SOC1 proteins. Two conserved regions were identified: MADS_MEF2_like domain and K-box domain. Amino acid sequence analysis and secondary structure analyses all revealed significant distinctions among the SOC1 proteins. Based on these amino acid sequence identities, the mango *MiSOC1* genes can be identified as belonging to the MADS-box family. MiSOC1 have a highly conserved MADS-box at the N-terminus and a K-box in the middle of the MADS-box and a highly conserved C-terminal motif, the SOC1 motif which are typical characters of MADS-box protein (Nakamura et al., 2005; Ruokolainen et al., 2011; Ding et al., 2013) (**Figure 1**). Analysis of predicted protein sequences classified MiSOC1 as MADS-box type II transcription factor. These data suggest that MiSOC1 belongs to MADS-box gene and is a putative SOC1 homolog in mango. A phylogenetic tree between SOC1 and SOC1-like subfamilies of MADS-box proteins was constructed showing the evolutionary relation verified MiSOC1 is high similarity with AGL20/SOC1 genes and classed into the AGL20/SOC1 group of dicots. The whole phylogenetic tree has family specificity and MiSOC1 was closely related to *Sapindaceae* SOC1. The sequence of MiSOC1 is most similar to SOC1 protein in *C. sinensis* (with 81% identity) and *L. chinensis* (with 84% identity). These data suggest that SOC1 might originate from the same ancestor, but evolve independently in dicots, monocots, gymnosperms.

The SOC1 genes have been widely isolated and characterized and the main function of SOC1 is as the integrator of multiple flowering signals. It also evolves to become functional divergence and is widely expressed in various tissues in plants (Li et al., 2008). It is speculated that SOC1 genes in are widely expressed in various tissues and have diversified regulatory functions in dicots plants (Borner et al., 2000). The characterization of mango MiSOC1 gene and analysis of it expression of different tissue, different varieties, reproductive development process and in response to plant growth regulators exogenous applications, will help in illuminating the molecular machinery in mango and other fruit trees. Previous studies have shown that SOC1 and SOC1-like genes are expressed in both reproductive and vegetative tissues (Lee et al., 2000; Nakamura et al., 2005; Papaefthimiou et al., 2012). MiSOC1 expression was detected in reproductive parts of mango at relatively low levels, including in flower bud, flower and fruitlet, whereas its expression in vegetative organs, such as root, stem and leaves significantly increased, with the highest level in leaves. Our study investigated a potential role of SOC1gene transform from vegetative development to reproductive development of mango and exogenous applications of plant growth regulators. As the vegetative phase in mango is usually a lengthy process that significantly affects the efficiency of mango breeding, identification of key flowering regulators such as MiSOC1 will contribute to improving mango flowering traits through either classical breeding or novel genetic engineering approaches.

In the present study, overexpression of MiSOC1 in *Arabidopsis* caused early flowering (**Figure 8A**) in agreement with previous studies (Shitsukawa et al., 2007; Ding et al., 2013; Heng-jiu et al., 2013; Shunli et al., 2015). MiSOC1 acted as a flowering activator in transgenic *Arabidopsis* which suggested that MiSOC1 plays an evolutionarily conserved role in the promotion of mango flowering and could have a positive role in regulation flowering of mango. To further understand the function of MiSOC1, we need to identify other genes or factors that are involved in the SOC1 integrated flowering pathway and investigate how these components control flowering in mango. Successful deciphering of the MiSOC1 genes will broaden our knowledge about the flowering control network in mango.

## Conclusion

In this study, we isolated and characterized the MiSOC1 with homology-based cloning and RACE. The open reading frame was 733 bps, encoding 223 amino acids with molecular weight 25.6 kD and isoelectric point 8.96. Sequence alignment and phylogenetic analysis indicated that it belonged to the SOC1/TM3 subfamily of the MADS-box family. The result of quantitative real-time PCR indicated MiSOC1 was widely expressed at different levels in both vegetative and reproductive organs and was fluctuated in diverse tissues/organs and developmental stages. In addition, MiSOC1 gene expression was affect by ethephon while high concentration ethephon inhibit the expression of MiSOC1. Overexpression of MiSOC1 in *Arabidopsis* caused early flowering suggests that MiSOC1 may act as induce flower function in mango. These findings increase our understanding of the mechanism of flowering control in mango.

## Author Contributions

JW and DL participated in experiment designing, data analysis, and drafting of the manuscript. GL collected materials and gave valuable advice on experiment preparation. JT performed gene clone and expression. YC took part in supervising the study. All authors read and approved the final manuscript.

## Conflict of Interest Statement

The authors declare that the research was conducted in the absence of any commercial or financial relationships that could be construed as a potential conflict of interest.

## Footnotes

1 http://www.ncbi.nlm.nih.gov/blast/Blast.cgi

2 http://myhits.isb-sib.ch/cgi-bin/motif_scan/

3 http://us.expasy.org/tools/peptide-mass.html

4 http://hits.isb-sib.ch/cgi-bin/PFSCAN

5 http://www.mbio.ncsu.edu/BioEdit/bioedit.html

6 http://www.ncbi.nlm.nih.gov/blast/Blast.cgi

7 http://www.cbs.dtu.dk/services/SignalP-4.0/
