# Supplementary figures and images for: Molecular Cloning, Characterization, and Expression of MiSOC1: A Homolog of the Flowering Gene SUPPRESSOR OF OVEREXPRESSION OF CONSTANS1 from Mango (Mangifera indica L)
Source: Front Plant Sci. 2016 Nov 29;7:1758. doi: 10.3389/fpls.2016.01758 (PMC5126060; doi:10.3389/fpls.2016.01758)

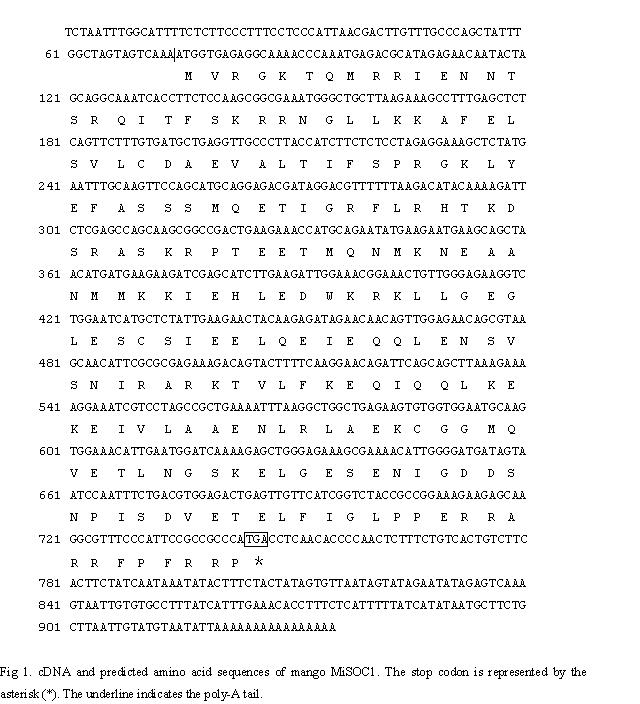

Supplement: Supplementary file 1 [file Image_1.JPEG]
